# Rapid bacterial community profiling of equine faecal, skin, milk and saliva samples using Oxford Nanopore long-read 16S rRNA amplicon sequencing

**DOI:** 10.1099/jmm.0.002176

**Published:** 2026-07-06

**Authors:** J. Leng, C. Tait, B. Alsubaie, A.H.M. Van Vliet, P. Sells, R.M. La Ragione, C Proudman

**Affiliations:** 1 School of Veterinary Medicine, Faculty of Health and Medical Sciences, University of Surrey, Daphne Jackson Road, Guildford GU2 7AL, UK; 2 Department of Clinical Sciences, College of Veterinary Medicine, King Faisal University, Al-Hofuf 36388, Saudi Arabia; 3 Chasemore Farm, Orbital Veterinary Services, Bookham Road, Downside, Cobham KT11 3JT, UK; 4 School of Biosciences, Faculty of Health and Medical Sciences, Edward Jenner Building, University of Surrey, Guildford GU2 7XH, UK

**Keywords:** amplicon sequencing, horse microbiome, Illumina sequencing, Oxford Nanopore sequencing, rapid diagnostics

## Abstract

**Introduction.** The composition of the equine gut microbiome is associated with many aspects of gastrointestinal, respiratory and musculoskeletal health that have been reported in the horse. Scientific studies exploring the microbiome non-intestinal ecological niches in or on horses are lacking. The clinical use of bacterial community profiling in horses is currently limited by cost and by slow analytical workflows.

**Hypothesis/Gap Statement.** Most equine microbiome studies have relied on 16S rRNA amplicon sequencing of bacterial DNA, using high-throughput short-read sequencing technologies. This is often provided by an external service due to the cost of Illumina and other sequencers. Analysis of such sequencing files relies upon the researcher to have prior experience of coding-based programs.

**Aim.** To explore the utility of Oxford Nanopore long-read sequencing in the analysis of microbiomes from several anatomical sites of the horse as a quicker and cheaper alternative to short-read sequencing.

**Methodology.** Bacterial DNA was extracted from horse (udder) skin swabs, saliva swabs, faecal samples and milk samples. Samples were prepared for Oxford Nanopore long-read sequencing and sequenced using a flow cell on the MinION Mk1D. Sequencing data were analysed using EPI2ME, along with extra analyses on exported taxa abundance data in R.

**Results.** Diversity measures and taxonomic relative abundance from phylum to family level were comparable to previously published equine studies that used Illumina sequencing. Sequencing data were acquired within 3 days costing around £30 per sample. Long-read sequencing gave accurate taxa assignment for two positive controls included at phylum, class, order and family levels of taxonomic classification.

**Conclusion.** This work demonstrates that long-read technologies such as Oxford Nanopore MinION sequencing can provide a reliable, quick and cost-effective alternative to short-read Illumina sequencing when characterizing microbial communities from a range of anatomical locations on/in the horse.

## Introduction

Over the last 15 years, there has been considerable research interest in the horse gut microbiome. Gut bacterial community profiles have been linked with multiple gastrointestinal diseases, including colitis, large colon volvulus and equine grass sickness [1,3]. Shifts in the equine gut microbiome have also been linked to diet [4,5], antibiotic use [6,7] and long-term health and performance outcomes [8]. Equine microbiome research has recently expanded beyond the gut to other organ systems. For example, the nasal and lung microbiomes have become a focus due to the potential of infectious respiratory diseases and equine asthma impacting horse performance [9]. In two recent studies, bacteria from the phylum *Proteobacteria* were identified as the most abundant in the respiratory microbiome [10,11]. Having a comprehensive understanding of the equine skin microbiome is also important because of the relatively high prevalence of skin disease in domestic horses [12]. Samples of the skin microbiome were found to have lower diversity and an increase in bacteria belonging to the *Staphylococcaceae* family in the areas of skin affected by pastern dermatitis [13]. The equine vaginal and uterine microbiomes have been studied to better understand associations with breeding and oestrous cycle. The vaginal and uterine microbiome of mares is characterized by the dominant phylum *Proteobacteria*, along with *Firmicutes*, *Bacteroidetes* and *Actinobacteria* also present in both microbiomes [14,17]. There are other microbiomes present in and on the horse such as that residing in the mouth that have not yet been fully characterized.

Most equine microbiome studies published to date have used high-throughput sequencing of a part of the bacterial 16S rRNA gene using high-throughput short-read sequencing such as that provided by the Illumina MiSeq platform. This is based on the amplification of hypervariable regions of the 16S rRNA gene followed by DNA sequencing to generate short-length reads. The choice of hypervariable region used is usually dependent on the type of sample being sequenced, and for example, the V4–V5 region is often amplified when analysing bacterial DNA extracted from faeces. Alternatively, the whole 16S rRNA gene can be amplified and sequenced, and this has been reported to be more accurate than sequencing single or multiple hypervariable regions of the gene [18]. Illumina sequencing technology relies on sequencing by synthesis of short DNA fragments, where fluorescent tags are cleaved when bases are added to single-stranded DNA which are then detected by the sequencer. Oxford Nanopore long-read sequencing utilizes protein pores that detect the change in electrical signal when single-stranded DNA or RNA passes through it [19] and does not require purchasing of expensive equipment, although running costs can be high depending on usage. Analysis of short-read Illumina sequencing data is computationally demanding, requiring advanced bioinformatics skills, extensive data processing and storage capacity. By contrast, data analysis of sequencing data generated by nanopore sequencing can be completed on the desktop program EPI2ME (Oxford Nanopore Technologies). Microbiome analysis has, therefore, been inaccessible for smaller, clinical laboratories and for researchers working outside of large university or commercial laboratories. Nanopore sequencing of the equine microbiome has the potential to reduce the cost, time and expertise historically needed for these analyses and could be integrated into veterinary clinical settings.

Our study reports the sequencing of the whole 16S rRNA gene from bacterial DNA extracted from samples collected from diverse anatomical locations on the horse using Oxford Nanopore Technology’s MinION platform. Our objectives were (i) to describe bacterial communities from diverse anatomical locations on the horse and (ii) to evaluate potential advantages and disadvantages of long-read nanopore portable sequencing technology for small veterinary clinic laboratories.

## Methods

### Equine sample acquisition

Fourteen samples were collected from horses on a Thoroughbred breeding stud in the south of England. A total of three skin swabs sampling the udder area were taken individually from three mares. Four saliva samples in total were taken, each swab from two mare and foal pairs. Four voided faecal samples were collected from mares, and two milk samples were collected from two lactating mares. Udder skin microbiome samples were collected by rubbing a dry transport swab (Vet Lab Supplies) on the skin of the mare’s teat for 30 s. Saliva was obtained using a PurSwab DNA-free Round Foam Swab (Puritan, USA), which was inserted into the interdental space of the horse’s mouth, above the tongue, and then removed after 30 s. Saliva swabs were placed in a sterile sample transport bag for storage. Freshly voided faeces were collected from the stable floor within an hour of defecation. Milk was manually expressed from the teats of the mares into sterile pots. All samples were transported to the laboratory at 4 °C and then frozen at −20 °C prior to DNA extraction. Information on all horses sampled can be found in Item S1, available in the online Supplementary Material.

### DNA extraction and MinION sequencing

DNA was extracted from all samples using the PureLink Microbiome DNA Purification kit (Thermo Fisher). This DNA kit has differing protocols depending on the sample type being used, and so these were followed with no modifications. However, there is no milk-specific protocol for bacterial DNA extraction for this kit; therefore, two milk samples were processed like saliva after a pellet was produced by centrifugation of 1.5 ml of milk (14,000 ***g*** for 2 min). To compare protocols, DNA was also extracted from 1.5 ml of whole milk (the same sample as used for milk pellet2 in Item S1) using the kit’s faecal DNA extraction method, which involves an additional clean-up step. This approach to extracting DNA from milk was similar to that reported by Barden *et al*. [20]. All DNA extracts were quantified using a Qubit dsDNA high-sensitivity assay kit and Qubit fluorometer (Thermo Fisher) and then stored at −20 °C until defrosted for sequencing. Along with the DNA extracts from the horse samples, two positive controls and a negative control were sequenced. These were a microbial community DNA standard (ZymoBIOMICS), a DNA extract from a single *Escherichia coli* isolate and nuclease-free water, respectively. DNA extracts were prepared for nanopore sequencing using the Rapid Sequencing DNA 16S Barcoding Kit 24 V14 (SQK-16S114.24, Oxford Nanopore Technologies) using the manufacturer’s instructions. This kit amplifies the whole of the 16S rRNA gene using the primers 27F and 1492R [21], along with adding 1 of 24 barcodes sequences to each sample so that up to 24 samples can be sequenced at once. Samples were sequenced on an R10.4.1 flow cell which was loaded into an mk1D sequencer (both Oxford Nanopore Technologies). The sequencing was carried out with a Dell Pro4 laptop which ran MinKNOW (version 25.05.12, Oxford Nanopore Technologies) with no base calling or splitting of sequences by barcodes occurring during the sequencing run.

### Analysis of sequencing data

After sequencing for 17 h, the resulting pod5 files were uploaded to the high-powered computer cluster hosted by the University of Surrey, and base calling was completed using Dorado (release 1.0.0, Oxford Nanopore Technologies). The bam files generated were split by the different barcodes added to the DNA from each sample using Samtools (version 1.21) to generate a single file for each of the samples analysed. Bam files were downloaded and analysed using the 16s workflow on EPI2ME (desktop version 5.2.5, Oxford Nanopore Technologies). During the setup of the workflow, KRAKEN2 was used for classification method, and the reference database ‘ncbi_16s_18s’ was chosen.

Alpha diversity measures and read counts were downloaded from EPI2ME at the species level. Diversity measures were saved as a text file, imported into R and visualized as boxplots using ggplot2 (version 3.5.2). Read count data were imported to R in .csv file format as a phyloseq object, along with a taxa and metadata file (both in .csv format). The R package phyloseq [22] (version 1.50.00) was used to calculate Bray–Curtis distances and draw a non-metric multi-dimensional scaling (NMDS) plot with these data. The package ANCOM-BC (Analysis of Compositions of Microbiomes with Bias Correction) [23] (version 2.8.1) was used to run the differential abundance analysis (using ANCOM-BC2) to identify taxa that differed between samples when grouped by sample type at phyla, class, order, family and genus levels of taxonomic classification. Differences identified using ANCOM-BC2 were visualized in a heatmap using this package, along with ggplot2 (version 3.5.2) and dplyr (version 1.1.4).

## Results

### Quick sample preparation and sequencing with Oxford Nanopore MinION

Samples were grouped by sample type and DNA extracted in separate batches for each sample type (a total of four batches), each of which took around 2 h. DNA was quantified after extraction demonstrating that saliva samples, on average, yielded the highest amount of DNA, whereas milk samples yielded the least (Table 1). The dilution of samples to 10 ng µl^−1^ followed by PCR to amplify the 16S rRNA gene and to attach sample-specific barcode sequences took around 3 h, of which the PCR cycles from the protocol represented 1 h 45 min. Addition of barcode sequences to each sample ensured that all 18 samples could be sequenced in parallel with reads from each sample separated subsequently by the barcode. The rest of the protocol was completed the following day, including the loading of the sample to the flow cell for sequencing, which took a further 2 h. MinION sequencing was left to run for 17 h (overnight).

**Table 1.** Quality control data from 15 unique equine biological samples of four types plus positive and negative controls, sequenced using Oxford Nanopore sequencing

Sample ID	DNA quantity (ng/µl)	Barcode	No. of reads	No. of reads unclassified	Percentage of reads unclassified
Skin1	0.52	BC01	34,798	123	0.35
Skin2	0.53	BC02	1,057	167	15.80
Skin3	0.58	BC03	27,023	107	0.40
Saliva1	0.27	BC04	108,176	157	0.15
Saliva2	8.78	BC05	107,175	354	0.24
Saliva3	6.24	BC06	86,728	137	0.16
Saliva4	64.00	BC07	153,818	337	0.22
Saliva5	52.40	BC08	146,160	244	0.17
Faeces1	21.9	BC09	58,826	117	0.30
Faeces2	4.60	BC10	20,410	101	0.49
Faeces3	31.40	BC11	92,543	232	0.25
Faeces4	37.60	BC12	117,782	270	0.23
Milk pellet1	0.15	BC13	1,669	86	5.15
Milk pellet2	< 0.1	BC14	1,116	78	6.99
Milk liquid	< 0.1	BC15	435	74	17.01
Positive community	10	BC16	143,366	185	0.13
Positive *E. coli*	1392	BC17	218,346	679	0.31
Negative	n/a	BC18	354	145	57.09

### Nanopore sequencing generated a high number of good-quality sequencing reads

**Fig. 1.**
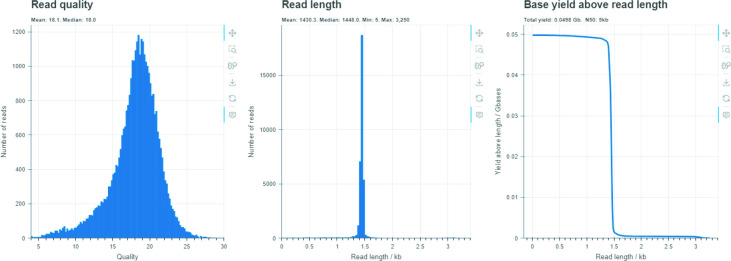
Quality control figures showing the read quality, length and base yield above read length from the report generated from EPI2ME analysis of skin sample 1. Read quality for most of the reads is high. Nearly all of the reads sequenced are 1,500 bp long which is the length of the whole 16S rRNA gene.

A total of 1,319,782 sequencing reads were generated from the 18 samples analysed: three skin, five saliva, four faecal and three milk samples, along with two positive controls and a negative control. The average number of reads generated per sample was 73,321. The DNA quantity along with the number of sequencing reads generated from each sample can be seen in Table 1. All samples generated >10,000 reads, apart from skin sample 2, the three milk samples and the negative control. Fig. 1 shows an example of the quality control data generated by EPI2ME for skin sample 1, showing that read quality was good and in a normal distribution. Reads were reported to be mostly 1,500 bp in length which is the length of the whole 16S rRNA gene.

### Microbiome diversity and compositional data can be derived from nanopore sequencing of equine samples

**Fig. 2.**
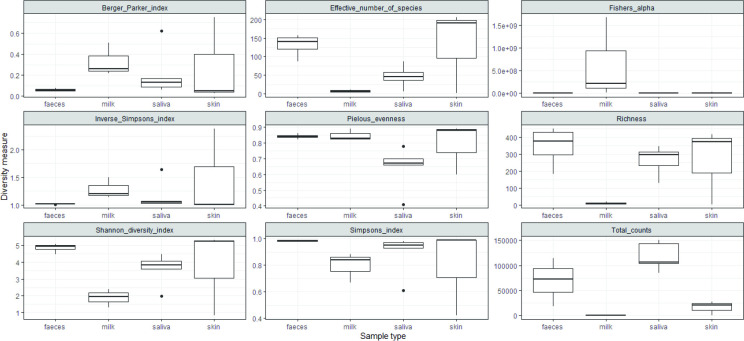
Alpha diversity measures and total counts generated by EPI2ME analysis of nanopore sequencing data from equine samples (*n*=15). These data were imported into R and visualized as boxplots with samples grouped by sample type using the package ggplot2.

Alpha diversity data generated from sequencing files give information on the bacterial species diversity of each sample analysed. Nine measures of alpha diversity were generated by the EPI2ME analysis of the sequencing files (Item S2), along with species richness curves (Item S3). These data showed that the full richness of the samples was captured at around 10,000 sequences, where the richness curves plateau. Diversity data were grouped by sample type and boxplots presented to assess difference in diversity between different sample types (Fig. 2). Faecal samples were found to have the highest bacterial diversity for several measures, including richness and Shannon diversity (a measure of community richness and evenness), whereas milk had the lowest diversity. However, skin samples had the highest diversity when measured as effective number of species.

**Fig. 3.**
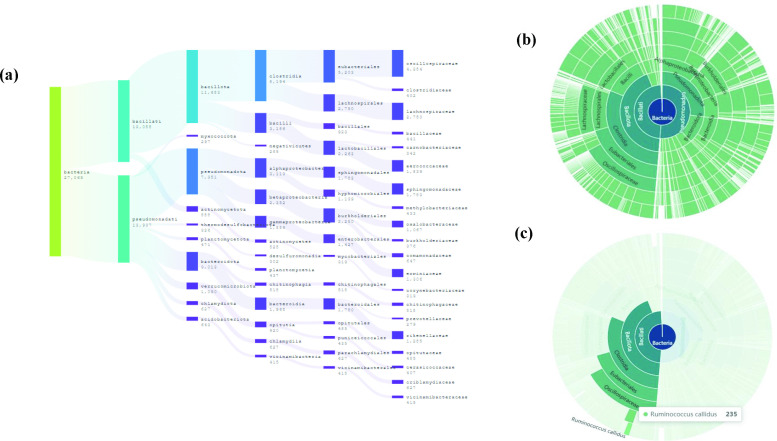
Example of different visualization of taxa relative abundance for skin sample1 from EPI2ME. (**a**) Lineages (numbers alongside the name of the taxa are the number of reads) and (b) sunburst plots showing the proportion of reads belonging to taxa at all levels of taxonomic classification. (**c**) Interactive sunburst plot with taxonomic lineage of *R. callidus* selected.

Community profile data for skin sample 1 are presented in Fig. 3, showing an example of the data presentation by EPI2ME. The lineage plot in Fig. 3(a) is created at the family level, although it can be drawn to the species level. The sunburst plot generated by EPI2ME (Fig. 3b) shows similar information to the lineage plot. The species *Ruminococcus callidus* was selected using the plot’s interactive feature so that its detailed taxonomy can be explored (Fig. 3c).

**Fig. 4.**
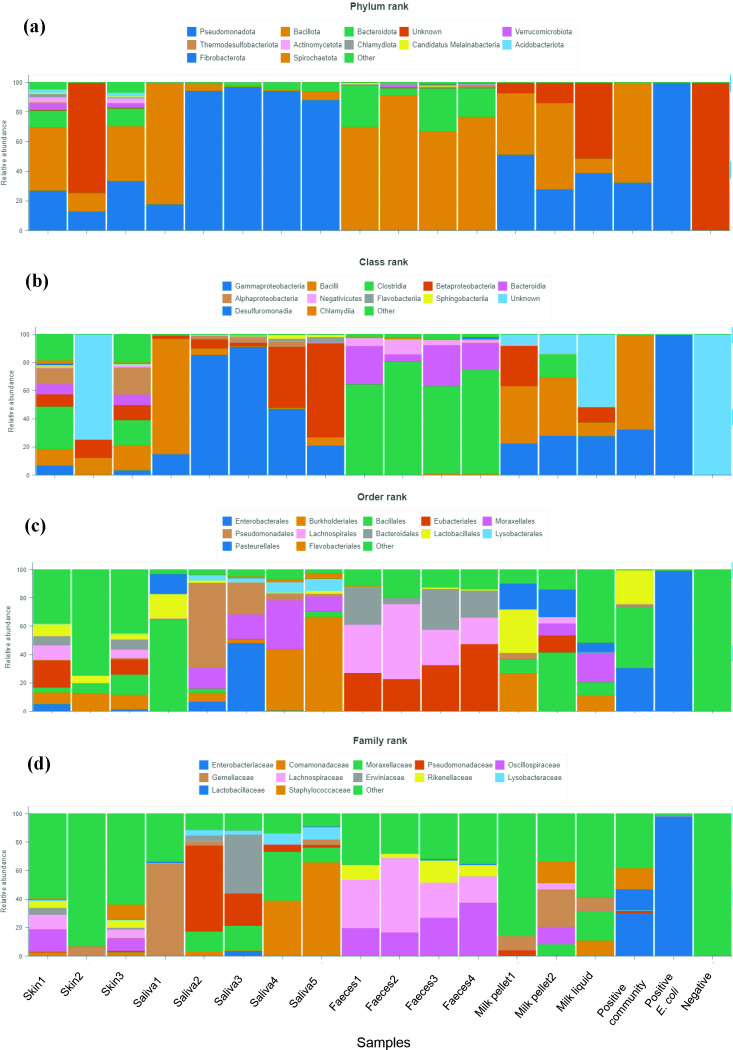
Relative abundance of taxa identified at (a) phylum, (**b**) class, (**c**) order and (d) family levels of taxonomic classification for all samples sequenced. The top 12 most abundant taxa across all samples are visualized with the relative abundance of the remainder classified as ‘Other’.

Percentage abundance plots for the 12 most abundant taxa are presented at phylum, class, order and genus levels (Fig. 4). These plots highlight the differences in the dominant phyla between samples from different anatomical locations. For example, at the phylum level, there is a large percentage of reads that belong to *Pseudomonadota* (formally known as *Proteobacteria*) in saliva samples, whereas the most abundant phylum in faecal samples is *Bacillota* (formally *Firmicutes*). At the family level, a large percentage of the reads were classified as ‘Other’ due to the diversity of families identified across sample types and the report only visualizing the 12 most abundant taxa. This figure also highlights samples ‘skin2’ and ‘milk liquid’ that have produced too few reads for taxa to be identified accurately, as many of the reads generated were designated as ‘unknown’ at all levels of taxonomic classification visualized.

### Importing MinION sequence data into R allows for further analyses and graphical presentation

**Fig. 5.**
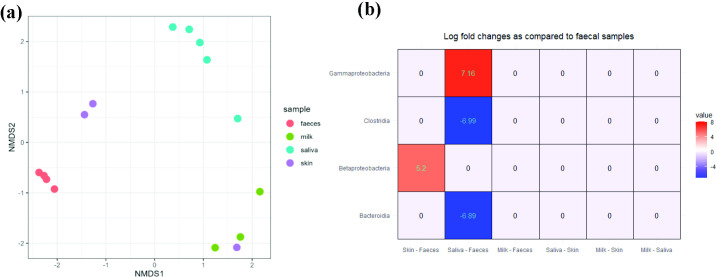
Further analysis of sequencing data after importing bacterial count data into R as a phyloseq object. (**a**) Beta diversity plot drawn after calculating Bray–Curtis distances. (**b**) Heatmap showing the bacterial classes identified as significantly differing in abundance between the groups of samples using ANCOM-BC2 on the class count data. The numbers in the heatmap indicate the log-fold change of the abundance of the bacterial class between the two groups. Colour indicates higher (red) or lower (blue) log-fold change in the first group compared to the second group in the x-axis legend.

Taxa abundance data generated by EPI2ME were imported into R along with a taxonomy file and a metadata file as a phyloseq object for further analyses. This allowed the Bray–Curtis dissimilarity data to be computed and an NMDS plot to be created with the data. This shows distinct clustering of samples linked to sample type (Fig. 5a); for example, all points representing faecal samples cluster together and away from other types of samples. ANCOM-BC2 was then used to identify taxa that differed in abundance between the types of samples, and a heatmap was drawn to visualize these differences (Fig. 5b). A separate ANCOM-BC2 analysis was run for phylum, class, order and family levels of taxonomic classification. At phylum level, two phyla were identified to differ significantly in abundance when comparing two groups at a time. Bacteria from the phylum *Pseudomonadota* were higher in abundance in both skin and saliva samples (4.57 and 6.39 log-fold difference, respectively) compared to faecal samples, but lower in milk samples compared to skin and saliva (3.1 and 4.92 log-fold difference). Bacteria from the phylum *Bacillota* were lower in abundance in milk samples compared to faecal and skin samples (4.65 and 3.59 log-fold change difference, Item S4). Four bacterial classes were identified as differing in abundance when comparing groups. Bacteria from the *Betaproteobacteria* class were 5.2 log-fold change higher in abundance in skin samples compared to faecal samples. For the comparison of saliva to faecal samples, *Clostridia* and *Bacteroidia* were 6.99 and 6.89 log-fold change (respectively) lower in abundance, and *Gammaproteobacteria* were 7.16 log-fold change higher in abundance (Fig. 5b).

### Analysis of controls shows lack of accuracy at genus and species levels

The bacterial genera and species identified from the microbial community DNA standard (positive control) using the nanopore sequencing and analysis were compared to the percentage abundance provided by the manufacturer for this standard (Table 2). At the genus level, four of the eight genera were identified at a percentage abundance that was expected or higher, whereas the remaining four were identified at a lower percentage. At the species level, only *Listeria monocytogenes* was identified at the expected abundance, and *Lactobacillus fermentum* was identified at a higher abundance than predicted. The remaining six bacterial species were detected at a lower percentage abundance than expected.

**Table 2.** Expected and observed percentage relative abundance of bacterial genera and species in the positive control. Expected abundance does not total 100%, as there were 4% eukaryotes (2% *Saccharomyces cerevisiae* and 2% *Cryptococcus neoformans*) in the expected abundance from the manufacturer, and analysis of eukaryotes was not included in this analysis

Genus	Expected abundance (%)	Observed abundance (%)	Percentage variance (%)
*Listeria*	12	12	0
*Pseudomonas*	12	2	−10
*Bacillus*	12	13	+1
*Escherichia*	12	<1	−12
*Salmonella*	12	9	−3
*Lactobacillus*	12	15	+3
*Enterococcus*	12	15	+3
*Staphylococcus*	12	9	−3
**Species**	**Expected abundance (%)**	**Observed abundance (%)**	**Percentage variance (%)**
*L. monocytogenes*	12	12	0
*Pseudomonas aeruginosa*	12	1	−11
*Bacillus subtilis*	12	<1	−12
*E. coli*	12	0	−12
*Salmonella enterica*	12	9	−3
*L. fermentum*	12	15	+3
*Enterococcus faecalis*	12	9	−3
*Staphylococcus aureus*	12	1	−11

## Discussion

In this study, we have demonstrated the utility of nanopore sequencing technology for bacterial microbiome sequencing in horses. We used this technology to acquire 16S rRNA gene sequence data from different ecological niches on/in the horse, and we demonstrated how this data can be used to infer bacterial community composition and have reported on data quality. MinION sequencing of 16S rRNA genes offers a rapid, relatively inexpensive route to bacterial community profiling with simplified hardware and data processing requirements. Sequencing data were generated in a maximum of 3 days of lab work at a cost of around £30 per sample. This cost was calculated by dividing the cost of the MinION mk1D sequencer starter pack by the maximum number of samples (a maximum of 144) that can be processed. For subsequent runs to this, only the flow cell and preparation kit would be needed, and therefore, the price per sample for subsequent runs would decrease further. We contend that this technology offers accessible research and clinical opportunities for equine veterinary medicine.

After DNA was extracted from samples with a commercially available kit, Oxford Nanopore Technologies’ sequencing preparation kit was used to prepare extracted DNA for sequencing. This kit can prepare up to 24 samples to be sequenced together and can be completed within 1 day, and the sequencing on the flow cell can be run overnight. This allows for rapid results such as those needed within a clinical setting. DNA extraction produced DNA of sufficient concentration for amplicon sequencing and read counts high enough for reliable data from all samples apart from the milk samples. Cow’s milk has previously been reported to have generated low sequencing reads [20], probably due to its low microbial load. Nanopore sequencing is being used within human clinical practice, including disease outbreak surveillance and microbiome profiling [24,25].

One of the barriers to timely results being generated by DNA sequencing is the bioinformatic analysis required to generate the desired data. DNA sequencing often generates large amounts of data, so command line-based programs and high amounts of computer power are required which demand the use of high-powered computer clusters. Oxford Nanopore Technologies has removed this obstacle by developing their own desktop application, EPI2ME, that can be used on Microsoft, Mac or Linux computers. This program can be run locally but also on the cloud which means large amounts of local computing power are not needed. Sequencing data are initially stored within pod5 files containing the changes in ionic current that occur as the DNA or RNA passes through the protein pores of the flow cell. These files must undergo ‘base calling’ to convert the electrical signal into DNA or RNA sequences in the form of fastq or bam files. Oxford Nanopore has developed its own base calling program, Dorado. This means that if the computer running the nanopore sequencing is powerful enough, the base calling can be run at the same time as sequencing using MinKNOW (Oxford Nanopore’s sequencing program). Alternatively, base calling can be performed using MinKNOW after the sequencing or by downloading Dorado to a Linux computer or high-powered computer cluster. Base calling using Dorado is also available within EPI2ME, but there is currently no cloud computing for this workflow. For this reason, we ran base calling on the high-powered computer cluster hosted by the University of Surrey.

The results report generated by the EPI2ME analysis of sequencing files included plots and data that described the bacterial communities of samples analysed. These were similar to those produced by QIIME2 [26] which is commonly used to analyse Illumina 16S rRNA amplicon sequencing. Although lineage and starburst plots were not necessary for this work, they illustrated the taxonomic lineages of bacteria identified. Our data from faecal samples show similar results to previous publications arising from Illumina sequencing, where two predominant taxa at phylum and class levels were observed: the phyla *Bacillota* and *Bacteroidota* and the classes *Clostridia* and *Bacteroidia* [27,28]. However, we acknowledge that we have not reported a direct comparison of Oxford Nanopore and Illumina sequencing of the same samples in this manuscript.

The horse salivary microbiome is currently unreported using 16S rRNA gene sequencing. Our analysis shows that it is dominated by bacteria belonging to the *Pseudomondota* phylum, but unsurprisingly, we encountered significant variation in class, order and family abundance. It is notable that within the two mare–foal dyads sampled for this study, the mare and foals had similar salivary microbiomes. Mare’s milk was also analysed for this study but generated low DNA quantities and reads number, so the results reported here may not be an accurate representation of the small number of bacteria present within the samples.

Data generated by EPI2ME can be imported into R as a phyloseq object for further analyses. This provides the opportunity to generate beta diversity plots and to undertake discriminant analyses to identify taxa that are significantly different between groups of samples. The NMDS plot drawn using a Bray–Curtis dissimilarity matrix shows differences between the samples taken, although we acknowledge that we have a relatively low number of samples per group in this study. This is not surprising as our samples were derived from different anatomical niches. ANCOM-BC2 analysis identified phyla and classes that differed between sample groups. However, no taxa were identified as differentially abundant at order, family and genus levels which was likely due to low sample numbers and therefore low power to detect differences between each group.

Zymobiomics microbial community DNA standard was included in the sequencing run to assess the accuracy of the Oxford Nanopore Technology’s 16S rapid barcoding kit and MinION sequencing. Although this method was relatively accurate at phylum, class, order and family levels of taxonomic classification, at genus and species level, not all taxa were identified. The only bacterial genus and species that were identified at the correct abundance were *Listeria* and *L. monocytogenes*. All others were identified at higher percentage abundance (three genera and one species) or lower (four genera and six species). The whole 16S rRNA gene of the DNA contained within the Zymobiomics microbial community DNA standard has previously been sequenced using the MinION platform by Zhang *et al.* [29]. They reported 80–90% correctly assigned reads at genus and species when analysing this sample. The observed differences in our data may be explained by our use of different analysis software and reference databases.

Although Oxford Nanopore sequencing offers a quicker and more cost-effective solution to microbiome profiling than other sequencing approaches, there are several barriers to its implementation within veterinary clinics, especially smaller practices. The main obstacle is access to computing power needed to be able to run the MinION sequencer and the subsequent base calling. Recommended computing requirements suggested by Oxford Nanopore include 32 GB of memory, 12 cores of CPU and 2 TB of storage, which is a higher specification than the average laptop. Base calling can be done after sequencing using the program MinKNOW, and the time required for this step is dependent upon the power of the computer. Secondly, our analysis of a microbial standard suggests that 16S amplicon sequencing using nanopore sequencing is not reliable to genus and species levels of taxonomic classification. Therefore, it should not be used to identify specific species but rather to provide an overview of a bacterial community.

## Conclusion

Our study demonstrates that 16S rRNA gene sequencing using the Oxford Nanopore Technologies’ MinION platform can be used for relatively rapid and simple bacterial community profiling in the horse. Sample collection was quick and non-invasive, and DNA could be extracted from all samples using the same extraction kit. Plots generated by EPI2ME are sufficient for exploration of bacterial diversity, and data can be downloaded and imported to R for further analysis. Data obtained from MinION sequencing were used to generate an overview of bacterial taxa to the family level. However, classification of our bacterial community DNA standard was not accurate at the genus or species level. We propose that Oxford Nanopore sequencing using the MinION platform, which produces data more rapidly and with lower computing power than other sequencing platforms, has the potential for use within small laboratories and in veterinary clinical settings.

## Supplementary material

10.1099/jmm.0.002176 Supplementary Material 1.

## Abbreviations

ANCOM-BC: Analysis of Compositions of Microbiomes with Bias Correction
NCBI: National Center for Biotechnology Information
NMDS: non-metric multi-dimensional scaling
QIIME: Quantitative Insights Into Microbial Ecology
