# Supplementary material for: Rapid bacterial community profiling of equine faecal, skin, milk and saliva samples using Oxford Nanopore long-read 16S rRNA amplicon sequencing
Source: J Med Microbiol. 2026 Jul 6;75(7):002176. doi: 10.1099/jmm.0.002176 (PMC13336655; doi:10.1099/jmm.0.002176)
Supplement: Supplementary Material 1. [file jmm-75-02176-s001.pdf]

Supplementary Material - Rapid bacterial community profiling of equine faecal, skin, milk and saliva samples using Oxford nanopore long-read 16S rRNA amplicon sequencing.

Supplementary item 1: Information on horses sampled (all thoroughbreds). All samples were collected from the same Thoroughbred racehorse stud.

Sample ID	Horse ID	Sex	Age
Skin1	1	F	8 years
Skin2	2	F	17 years
Skin3	3	F	11 years
Saliva1	4	F	10 years
Saliva2	5	Mare1 F	7 years
Saliva3	6	Foal1	2 days
Saliva4	7	Mare2 F	8 years
Saliva5	8	Foal2	2 days
Faeces1	9	F	15 years
Faeces2	10	F	8 years
Faeces3	9	F	15 years
Faeces4	11	F	10 years
Milk pellet1	9	F	15 years
Milk pellet2	12	F	10 years
Milk liquid	12	F	10 years

Supplementary Item 2: Alpha diversity measures for all samples analysed.

	Skin1	Skin2	Skin3	Saliva1	Saliva2	Saliva3	Saliva4	Saliva5	Faeces1	Faeces2	Faeces3	Faeces4	Milk Pellet1	Milk Pellet2	Milk Liquid	Positive community	Positive *E. coli*	Negative control
**Berger Parker**	0.03	0.75	0.05	0.62	0.06	0.17	0.09	0.13	0.05	0.06	0.05	0.08	0.22	0.26	0.51	0.15	0.33	1
**Effective number of species**	205.8	2.29	189.96	7.29	86.69	46.29	58.7	36.36	131.61	87.4	157.57	148.62	10.88	7.13	3.76	29.06	12.14	1
**Fisher's alpha**	401.99	2.6 x 10^7	440.88	287.48	288.36	301.38	268.51	270.95	330.38	457.49	299.22	283.06	1.7 x 10^9	2.1 x 10^8	1.3 x 10^7	271.6	251.28	1.3 x 10^7
**Inverse Simpson's index**	1.01	2.38	1.01	1.64	1.02	1.07	1.03	1.05	1.02	1.02	1.01	1.02	1.14	1.2	1.5	1.08	1.2	none
**Pielou's evenness**	0.88	0.6	0.89	0.41	0.78	0.67	0.7	0.66	0.84	0.86	0.84	0.82	0.83	0.89	0.82	0.6	0.51	0
**Richness**	415	4	374	128	296	313	346	232	332	182	422	450	18	9	5	277	136	1
**Shannon index**	5.32	0.83	5.25	1.99	4.46	3.83	4.07	3.59	4.88	4.47	5.06	5	2.39	1.96	1.32	3.37	2.5	0
**Simpson's index**	0.99	0.42	0.99	0.61	0.98	0.93	0.97	0.95	0.99	0.98	0.99	0.98	0.88	0.84	0.67	0.92	0.83	0
**Total counts**	27,192	224	20,400	106,067	104,484	84,588	150,606	150,606	143,532	56,386	87,482	114,579	1,192	564	144	141,726	217,170	145

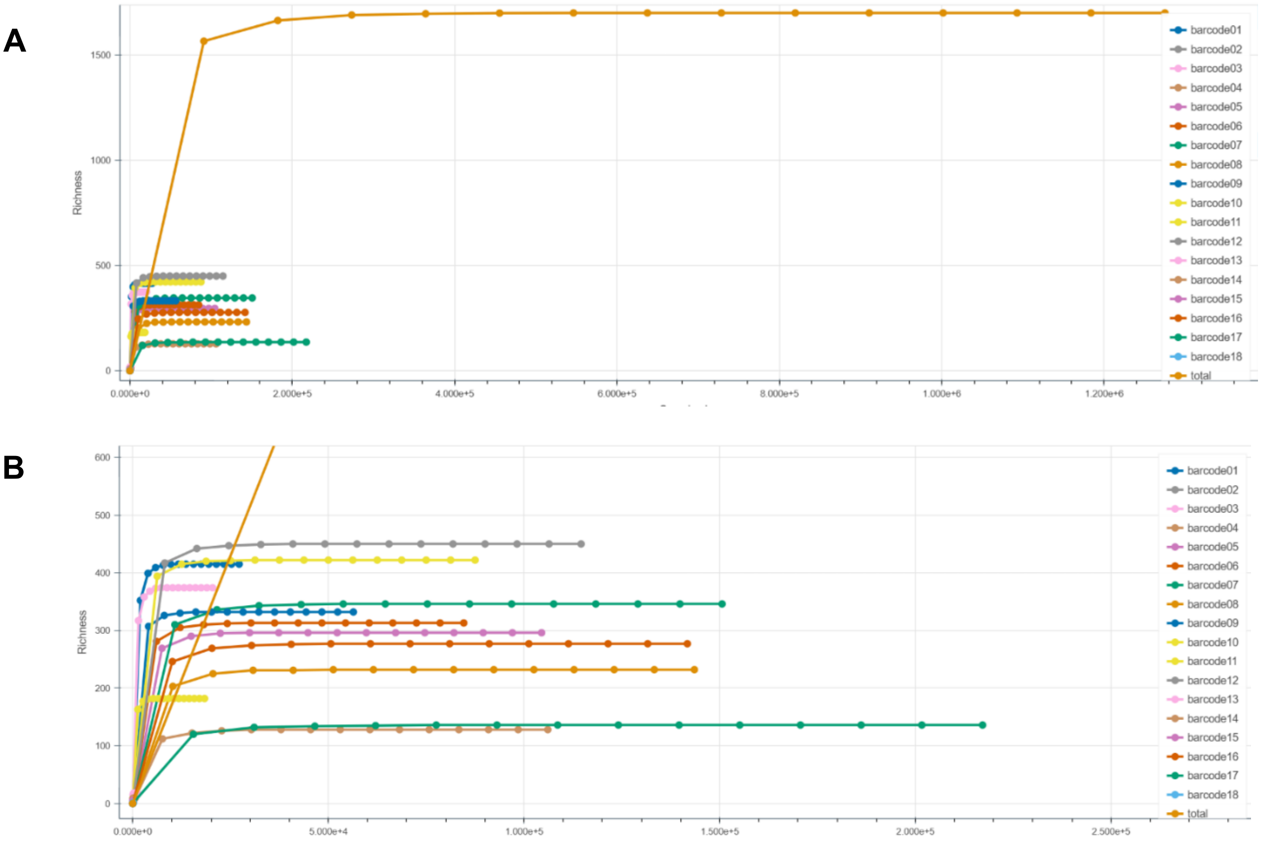

Supplementary Item 3: Species richness curves provided by the EPI2ME 16S sequencing report. A) All samples including richness calculated for all samples totalled and B) zoomed in to show detail of the samples analysed

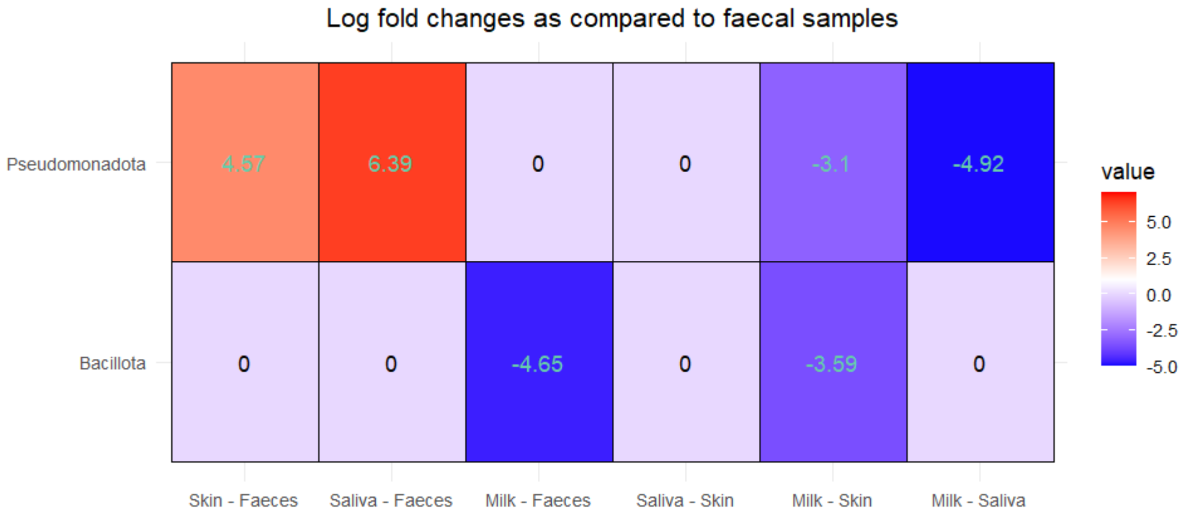

Supplementary Item 4: Heatmap showing the bacterial phyla identified as significantly differing in abundance between the groups of samples using ANCOMBC2 on the phyla count data. The numbers in the heatmap indicate the log foal change difference in abundance of the bacterial phyla between the two groups. Colour indicates higher (red) or lower (blue) log foal change in the first group compared to the first group in the x axis legend.
